# Mesenchymal stem cells: key players in cancer progression

**DOI:** 10.1186/s12943-017-0597-8

**Published:** 2017-02-01

**Authors:** Sarah M. Ridge, Francis J. Sullivan, Sharon A. Glynn

**Affiliations:** 1 Discipline of Pathology, Lambe Institute for Translational Research, School of Medicine, Costello Road, Galway, Ireland; 2 Prostate Cancer Institute, School of Medicine, Costello Road, Galway, Ireland

**Keywords:** Mesenchymal stem cells, Tumour microenvironment, Cancer progression, Tumour metastasis, Tumour stroma

## Abstract

Tumour progression is dependent on the interaction between tumour cells and cells of the surrounding microenvironment. The tumour is a dynamic milieu consisting of various cell types such as endothelial cells, fibroblasts, cells of the immune system and mesenchymal stem cells (MSCs). MSCs are multipotent stromal cells that are known to reside in various areas such as the bone marrow, fat and dental pulp. MSCs have been found to migrate towards inflammatory sites and studies have shown that they also migrate towards and incorporate into the tumour. The key question is how they interact there. MSCs may interact with tumour cells through paracrine signalling. On the other hand, MSCs have the capacity to differentiate to various cell types such as osteocytes, chondrocytes and adipocytes and it is possible that MSCs differentiate at the site of the tumour. More recently it has been shown that cross-talk between tumour cells and MSCs has been shown to increase metastatic potential and promote epithelial-to-mesenchymal transition. This review will focus on the role of MSCs in tumour development at various stages of progression from growth of the primary tumour to the establishment of distant metastasis.

## Background

It is now understood that tumour cells do not act alone. Cancer cells interact with their surrounding stroma and these interactions lead to an ‘activated state’ resulting in increased release of pro-inflammatory cytokines and growth factors [1]. The tumour is in a chronic state of inflammation and has been described as a ‘wound that never heals’ [2]. This inflammatory state drives the recruitment of responsive cell types such as macrophages, myeloid derived suppressor cells and mesenchymal stem cells (MSCs) [3–5]. Cross-talk between cancer cells and cells of the surrounding stroma promotes tumour progression and creates a dynamic extracellular matrix, favourable for the invasive tumour cell [6, 7].

The tumour stroma varies between each cancer type and the heterogeneous nature of the tumour makes it complicated to study. It is important to develop an understanding of what drives non-cancerous cells toward an activated state, what that activated state is and what it subsequently means for tumour cell progression.

MSCs are multipotent stem cells originally found to have the capacity to differentiate into the tri-lineages - osteoblasts, chondrocytes and adipocytes [8]. They are generally characterised by their tri-lineage differentiation capacity and by positivity for surface markers CD73, CD105 and CD90 [9]. More recent developments have revealed a wider range in differentiation potential such as differentiation to myocytes and neurons [10, 11]. They can be sourced from the bone marrow, adipose tissue and dental pulp [8, 12–14]. They are also found in circulation and are known to home to inflammatory sites [15]. Due to their capacity to home to injured tissue, research has suggested a reparative function for MSCs in multiple tissues including the lung [16], liver [17], brain [18] and heart [19].

MSCs reside in the bone marrow stroma alongside haematopoietic stem cells (HSCs), osteoblasts, osteoclasts, adipocytes, endothelial cells (ECs) and monocytes [20, 21]. MSCs may play a supportive role for HSCs and have previously been used to enhance long-term HSC engraftment in human transplantation [22, 23].

Knowledge of these characteristics as well as their differentiation capacity has caused excitement in the field of regenerative medicine and use of MSCs has potential for therapeutics in a range of fields such as cardiology, immunology and neurology. However, in the field of cancer research many studies suggest that MSC activity may contribute to poorer outcomes [24–27].

In recent studies, it has been shown that MSCs can also home to tumour sites and contribute to tumour growth and progression [26–29]. Analysis from human prostatectomies showed that MSCs represented 0.01–1.1% of total cells present in the prostate tumour [30]. MSCs have been found to increase the metastatic potential of tumour cells by promoting their motility and invasiveness as well as having a role in the creation of a metastatic niche at the secondary site [26, 31–33].

## Main text

### Mesenchymal stem cells at the primary tumour site

MSCs have been implicated in the promotion of tumour growth in numerous cancer types such as follicular lymphoma [24], head and neck carcinoma [25], glioma [34], breast [26], gastric [35], colon [36] and prostate cancer [27].

Karnoub and colleagues showed that co-injection of human bone marrow MSCs with only one of four breast cancer cell lines (MCF7) into mice led to accelerated tumour growth, however, co-injection with all cell lines (MDA-MB-231, HMLR, MDA-MB-435 and MCF7) led to increased metastasis [26]. Similarly, in a more recent study it was found that co-injection of human bone marrow MSCs with the triple negative inflammatory breast cancer cell line, SUM149, resulted in inhibited primary tumour growth but increased invasion and metastasis in mice [37]. These findings indicate a role for MSCs at the tumour site in the promotion of metastasis possibly through the induction of epithelial-to-mesenchymal transition (EMT) in primary tumour cells.

**Fig. 1:**
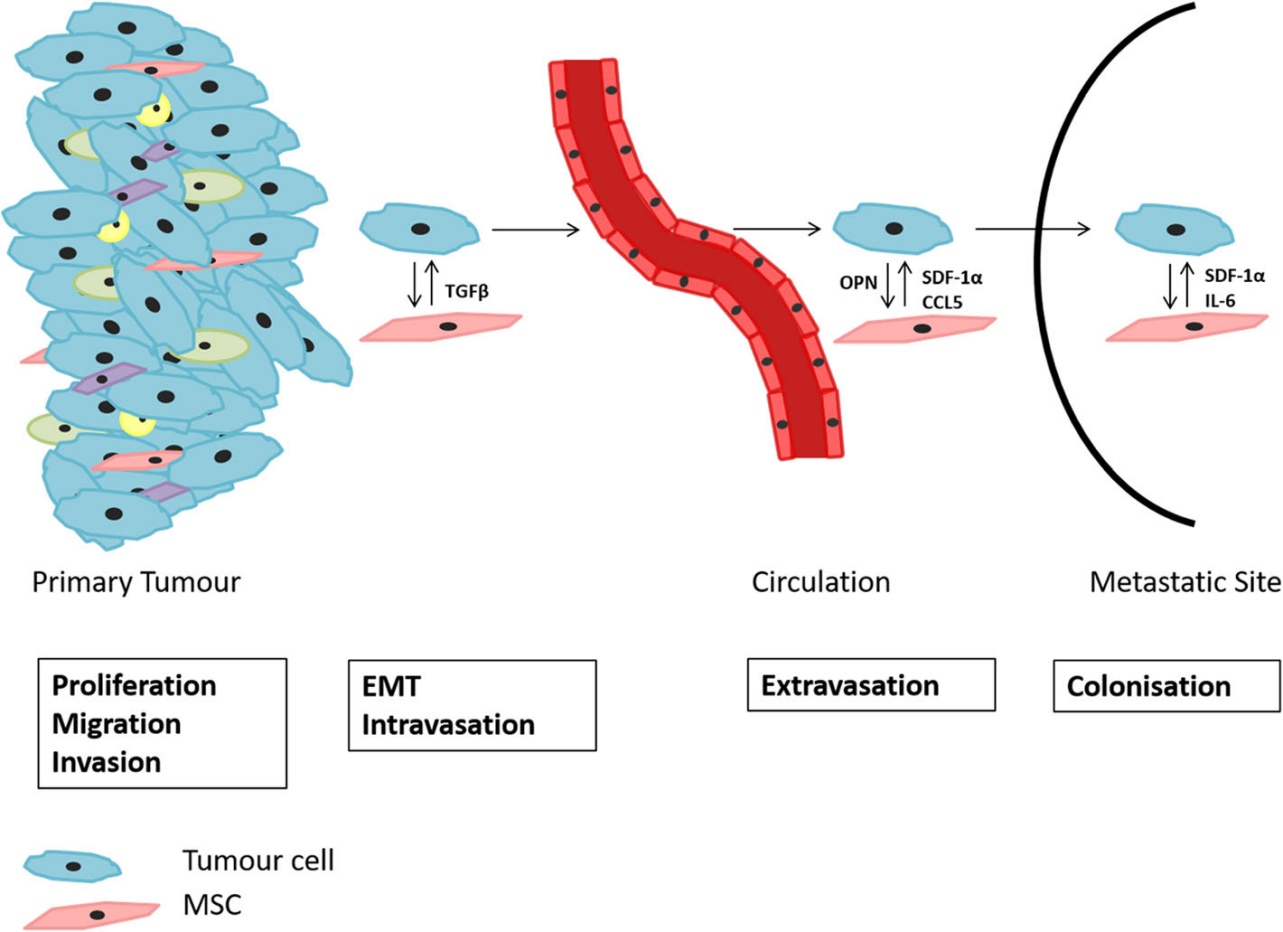
MSC and tumour cell interaction in cancer progression. MSCs have been shown to interact with tumour cells at the primary site and during metastatic colonisation in a manner that promotes cancer progression. MSCs have been shown to promote EMT in tumour cells through direct cell-cell contact, which could in part be due to TGFβ secretion [38, 82]. Additionally, tumour cell secretion of osteopontin (OPN) was found to induce MSC secretion of chemokine (C-C motif) ligand 5 (CCL5) stimulating breast cancer cell metastasis through interaction with the C-C chemokine receptor type 5 (CCR5) receptor [84]. Tumour cell migration towards and entry into the bone marrow metastatic site was shown to be mediated by stromal cell-derived factor 1 (SDF-1α) – a factor secreted by bone marrow MSCs – interaction with the C-X-C chemokine receptor type 4 (CXCR4) receptor expressed on breast and prostate tumour cells [33, 102, 103]

An increase in tumour growth was also found in mice following co-injection of human adipose tissue derived MSCs with the prostate cancer cell line MDA-PCa-118b [27]. In another study bone marrow MSCs were also found to stimulate the proliferation, migration and invasion of the prostate cancer cell line PC3 in vitro (see Fig. 1). This effect was inhibited by blocking transforming growth factor β (TGFβ) [38]. A similar study showed that TGFβ immunodepletion from oncostatin M treated human adipose tissue derived MSC conditioned media reduced the adhesion capacity of PC3 cells in vitro [39]. Like many growth factors and cytokines TGFβ plays a dual role in cancer. TGFβ can have a suppressive effect during the early initiating steps of carcinogenesis, acting as a tumour suppressor inhibiting cell proliferation, while in later stages it can induce epithelial to mesenchymal transition promoting the development of metastatic disease [40]. Of particular note is the dependency on stromal derived TGFβ for colorectal cancer metastasis initiation [41], and the association of stromal TGFβ expression with breast cancer outcome [42].

Some research groups have investigated the tumour promoting function of MSCs isolated from the tumour, arguably a more realistic approach to understanding the role of MSCs within the tumour microenvironment. Co-injection of MSCs isolated from human head and neck carcinoma [43], gastric cancer [25] and gliomas [34] with tumour cells into mouse models resulted in an increase in tumour growth and progression. Interestingly, Li and colleagues found that MSCs isolated from human gastric cancer tumours stimulated increased proliferation and migration of gastric cancer cell lines (BGC-823 and MKN-28) in vitro in comparison to bone marrow derived MSCs or MSCs isolated from non-cancerous adjacent tissue. They also found that they secreted more vascular endothelial growth factor (VEGF), macrophage inflammatory protein-2, TGF-β1, and the pro-inflammatory cytokines interleukin (IL)-6, and IL-8 while blockade of IL-8 attenuated the tumour promoting function of the gastric cancer MSCs [35].

From the studies described thus far, we can ascertain that MSCs are important players in the promotion of tumour growth and progression. Key thoughts to consider at this point would be whether naïve MSCs can induce such an effect upon arrival at the tumour through paracrine signalling and cell-cell contact, or do tumour microenvironment exposed MSCs transition to a determined ‘activated’ or reprogrammed state. The studies discussed above describing MSCs directly isolated from the tumour give evidence to the latter whereby tumour derived MSCs differed in activity to naïve bone marrow derived MSCs. Taking this into account, future studies should consider further investigation into the functional and molecular differences that occur in MSCs isolated from various tumour types. Are they functionally, morphologically and molecularly the same or does it depend on the tumour source?

### Role in tumour suppression

In contrast to the research described above there is evidence to suggest that MSCs can also have an inhibitory effect on tumour growth. Suppression of tumour growth has been noted in breast cancer [44], Kaposi’s sarcoma [45], hepatoma [46] and melanoma [47] models. Human MSCs derived from the umbilical cord and adipose tissue were implanted into a breast cancer metastasis mouse model and found to inhibit metastasis to the lung and reduce tumour growth through poly (ADP-ribose) polymerase (PARP) and caspase-3 cleavage, which could in turn induce apoptosis [44]. However, MSCs derived from the bone marrow, adipose tissue and dental pulp are not functionally identical, therefore the studies using MSCs derived from other sources may not be replicated using bone marrow derived MSCs [48, 49].

MSCs are a heterogeneous population of cells containing subpopulations with differing differentiation capacities [50]. Moreover, MSCs were found to express embryonic stem cell or pluripotency markers which differed depending on the source. Bone marrow derived MSCs were found to express Oct4, Nanog, alkaline phosphatase and SSEA-4; adipose and dermis derived MSCs were found to express Oct4, Nanog, SOX2, alkaline phosphatase and SSEA-4; while heart MSCs were found to express Oct4, Nanog, SOX2 and SSEA-4 [51]. It is therefore relevant to consider the source of MSCs and the techniques used to isolate and characterise them in each study. Table 1 highlights the experimental methods used to identify MSCs in key studies described in this review. There is an apparent discrepancy between studies in the techniques used to isolate the MSCs, where only a portion used gradient centrifugation to separate a population of MSCs. Moreover, each study uses a different set of criteria to characterise the isolated population. Though the predominant positive markers used are CD105 and CD90, there is no overall consistency in molecular or phenotypic characterisation of the MSCs used in each study. Differences in isolation techniques and growth conditions can favour certain subpopulations and future research in this area should place emphasis on the methods for isolation and characterisation for increased clarification on the population of stromal cells used experimentally.

**Table 1:** Isolation techniques and methods of characterisation used in a selection of studies

Manuscript	Origin	Species	Isolation Technique	Characterisation	Tumour of Relevance	Tumour Function
Karnoub et al., 2007 [26]	Bone marrow (hip)	Human	Histopaque density centrifugation, bFGF supplemented, adherent to plastic	CD105^+^, CD45^−^/GlyA^−^	Breast	Promoting
Lacerda et al., 2015 [37]	Bone marrow	Human	Purchased from EMD Millipore (Billerica, MA, USA) (Part #SCC034, Lot N61710996)	Markers unspecified. Osteogenic, adipogenic and chondrogenic differentiation capacity.	Breast	Promoting
Ye et al., 2012 [38]	Bone marrow (iliac crest)	Human	Percoll gradient centrifugation, adherence to plastic	CD105^+^, CD90^+^, CD44^+^, CD29^+^, CD166^+^, HLA-ABC^+^, CD34^−^, CD14^−^, CD45^−^ and HLA-DR^−^. Osteogenic and adipogenic differentiation capacity	Prostate	Promoting
Lee et al., 2013 [39]	Adipose tissue	Human	Adherence to plastic	CD105^+^, CD90^+^, CD44^+^, CD29^+^, CD73^+^, CD34^−^, CD45^−^ and CD31^−^	Prostate	Promoting
Sun et al., 2009 [44]	Umbilical cord	Human	Ficoll density gradient centrifugation, adherence to plastic	CD105^+^, CD90^+^, CD44^+^, CD29^+^, CD73^+^, CD34^−^, CD45^−^ and HLA-DR^−^.	Breast	Suppressive
Sun et al., 2009 [44]	Adipose tissue (mammary fat)	Human	Adherence to plastic	Characterised in a previous study: CD105^+^, CD90^+^, CD29^+^, CD34^−^, CD14^−^, CD45^−^, HLA-DR^−^and CD133^−^.	Breast	Suppressive
Otsu et al., 2009 [47]	Bone marrow	Rat and mouse	Adherence to plastic	CD90^+^, CD44^+^, CD29^+^, CD59^+^, CD54^+^, CD11b^−^, CD45^−^	Melanoma	Suppressive when administered at a 3:1 ratio with ECs.
Spaeth et al., 2009 [61]	Bone marrow	Human	Adherence to plastic	CD105^+^, CD90^+^, CD44^+^, CD146^+^, CD140b^+^, CD166^+^, CD31^−^, CD34^−^and CD45^−^. Osteogenic, adipogenic and mineralised cell differentiation capacity	Transition to CAF following exposure to ovarian cancer ‘SKOV-3’ cells	Promoting following transition to CAF
Mishra et al., 2008 [70]	Bone marrow	Human	Ficoll gradient centrifugation, adherence to plastic	CD105^+^, CD90^+^, CD44^+^, HLA-ABC^+^, Stro1^+^, CD11b^−^, CD45^−^ and HLA-DR^−^. Osteogenic, adipogenic and myogenic differentiation capacity	Transition to CAF following exposure to breast cancer ‘MDA-MB-231’ cells	Promoting following transition to CAF
Shangguan et al., 2012 [65]	Bone marrow	Human	Obtained from IH-supported MSC Distribution center in Texas A&M Health Science Center	CD105^+^, CD90^+^, CD44^+^, CD29^+^, CD49c^+^, CD49f^+^, CD59^+^, CD166^+^, CD34^−^, CD36^−^, CD117^−^ and CD45^−^. Osteogenic, adipogenic and chondrogenic differentiation capacity	TGF-β dependent transition to CAF following exposure to breast cancer ‘MDA-MB-231’ cells	Promoting following transition to CAF

Otsu et al. showed that murine bone marrow MSCs had a cytotoxic effect on the tumour in a melanoma mouse model through the release of reactive oxygen species when in contact with ECs present at the capillaries. This induced apoptosis of the ECs and reduced tumour growth. However, the cytotoxic effect of the MSC was only observed when implanted at high concentrations. MSCs seeded onto EC derived capillaries in matrigel evoked a cytotoxic effect at a EC:MSC ratio of 1:1 or 1:3. Cytotoxicity decreased when the MSC number was reduced by an order of magnitude [47] and given that in prostate cancer MSCs were only found to represent 0.01–1.1% of the tumour experiments using a high ratio of MSCs may not be reflective of the tumour microenvironment in vivo [30]. These results may explain the difference in outcome observed in studies showing tumour growth promotion by MSCs. Further investigation on the effect of dose on efficacy is warranted for any conclusions to be made, nonetheless, when examining the impact of MSC on tumour biology, the source and specific ratios of MSC to tumour cells reflective of the natural tumour environment is an important consideration.

Another explanation for the contrasting results is that like macrophages there is a polarisation of MSCs in response to secreted factors from the tumour that either drives the cells toward a tumour promoting or suppressive function. Tumour infiltrating macrophages can become induced by the stromal microenvironment and are referred to as tumour associated macrophages (TAMs) [52, 53]. Depending on the stimuli, macrophages can be polarised toward an M1 or M2 phenotype. The M1 phenotype can be induced by interferon gamma (IFN-γ) and lipopolysaccharides and have been shown to have cytotoxic effects on tumour cells. In contrast M2 macrophages are induced by IL-4, IL-13 and IL-10, promote wound healing and angiogenesis and are phenotypically similar to TAMs [52, 54–56].

MSCs were previously found to express toll-like receptor (TLR)﻿- 1, 2, 3, 4, 5 and 6 and TLR-agonist interaction stimulated MSC migration and immunomodulatory factor secretion [57]. In particular LPS stimulation of TLR4 and Poly-IC stimulation of TLR3 resulted in enhanced phospho-IKKα/β and phospho-MAPK indicting that activation of TLR4 or TLR3 may regulate NFkB and/or MAPK signalling in MSCs. In particular IL-6 and IL-8 were highly induced upon TLR4 activation [57]. Interestingly, Waterman and colleagues proposed a polarisation of MSCs based on TLR signalling. They found functional differences between human bone marrow derived MSCs stimulated by either TLR4 or TLR3 and classified them as MSC1 and MSC2 respectively [58]. MSC1 cells were found to have an anti-tumour effect while MSC2 cells promoted tumour growth and metastasis [59]. Given that increased expression of both TLR3 and TLR4 in breast tumour epithelium is associated with increased risk of disease recurrence [60], and taken in the context of their anti-tumoural and pro-tumoural effects in MSCs [59], it is clear that targeting TLRs for the treatment of cancer is complex and its benefits may be dependent on the specific polarisation of MSCs and immune cells in the tumour microenvironment, in addition to the TLR expression patterns within the tumour epithelia in each individual patient.

### Cancer associated fibroblasts: origins and characteristics

Cancer associated fibroblasts (CAFs) are a heterogeneous population of fibroblast-like cells with a tumour promoting function. The heterogeneity may be due to varying cell origins and the molecular constitution of tumour stroma from which the cell fate is determined. CAFs have been found to originate from bone marrow MSCs, fibroblasts and by transdifferentiation of epithelial and endothelial cells [61–63]. The mechanisms by which the cells differentiate or become ‘activated’ are largely unknown, however, exposure to TGF-β has been shown to induce the phenotypic changes regardless of cell origin [63–66].

### MSCs as an origin for CAFs

Evidence to suggest CAFs can be derived from MSCs was found in in vivo studies whereby genetically tagged bone marrow derived cells, injected into mice, were found at the tumour site with myofibroblast morphology and expressing α smooth muscle actin (α-SMA) and the α_1_ chain of type I (pro)collagen [67–69]. A subsequent study in a murine ovarian carcinoma xenograft model, found that bone marrow derived MSCs engrafted at the tumour expressed CAF markers fibroblast activation protein, fibroblast specific protein 1, α-SMA and tenascin C (TN-C) [61].

Further evidence to support the hypothesis that CAFs can originate from MSCs comes from in vitro studies where MSCs are cultured long-term in tumour cell conditioned medium. In a study by Mishra et al. human MSCs were cultured for up to 30 days in the breast cancer cell line (MDA-MB-231) conditioned medium [70]. The resulting MSCs expressed increased levels of α-SMA, fibroblast specific protein 1 (FSP-1), SDF-1α and vimentin and stimulated tumour cell growth in both in vitro and in vivo models [70]. Long-term culture of human MSCs for 12–16 days in conditioned medium taken from ovarian cancer cell line, SKOV-3, induced the expression of CAF markers in MSCs and elevated secretion of IL-6, leading to increased tumour cell proliferation [61]. Interestingly, TGF-β may be involved in the transition as human bone marrow MSCs transduced with a lentiviral vector which inhibited TGF-β/smad signalling, expressed a decrease in CAF markers when conditioned for 10 days in tumour cell conditioned medium in comparison to naïve MSCs [65]. Furthermore, treatment of MSCs with the endoplasmic reticulum chaperone, GRP78, activated TGF-β/smad signalling and induced the transition to a CAF like phenotype [71]. Taken together, it is clear that TGF-β plays a major role in the transition from MSC to CAF, however it is unclear to what degree it affects the secretory profile of the cells and their functional characteristics. It is also interesting to note that the MSCs used in each of these studies are positive for the MSC markers CD105, CD90 and CD44, which allows a more robust interpretation of the findings (see Table 1).

On the other hand, it must be noted that MSCs and CAFs share many similarities. A study has shown that CAFs share many of the same surface markers as MSCs such as CD29, CD44, CD73, CD90, CD106 and CD117, and have the capacity to differentiate to osteocytes, chondrocytes and adipocytes, and express vimentin [72]. An interesting suggestion, which is discussed in more detail in a recent review by Kalluri, describes the idea that fibroblasts are resting mesenchymal cells that can be activated to become MSCs in response to certain stimuli [73]. Nonetheless, CAFs were found to have an increased proliferative capacity and secrete increased VEGF, TGF-β, IL-4, IL-10 and tumour necrosis factor-α (TNF-α) compared to MSCs [72]. This provides credibility to another proposal by Kalluri that resting fibroblasts are in fact MSCs that can be stimulated to an activated state such as what is described as a CAF or a cancer-associated MSC [73]. It could also be suggested that CAFs originate from a subpopulation of MSCs, a finding which could explain some of the shared characteristics. It was suggested in a review by Augsten that the term CAFs should be used to describe a heterogeneous population of fibroblasts that originate from different sources, reside in various tumour types but are not assigned a specific function. This suggestion borrows from previous literature describing macrophage polarisation where an F1 subtype would be associated with tumour suppressive properties and an F2 subtype would describe fibroblasts with tumour promoting effects [74].

### Mesenchymal stem cells and metastasis

MSCs interact with cancer cells at multiple stages of cancer progression. At the primary tumour MSCs have been shown to drive tumour cells toward an invasive, pro-metastatic state. Human MSCs injected alone into mice with mammary carcinoma xenografts resulted in a 42% occurrence of metastatic lesions, compared with 17% in the control treated mice [75]. Similarly, human MSCs injected systemically into mice were found to migrate to the stroma of primary colon tumours as well as metastatic liver tumours [76]. Furthermore, co-culture of human bone marrow MSCs with MDA-MB-231 or MDA-MB-435 breast cancer cell lines 48 hours preceding injection resulted in enhanced metastasis in a mouse orthotopic implantation model, whereas the MSCs had no effect on metastasis without prior co-culture [77].

Tracking of MSCs using magnetic resonance imaging in a mouse xenograft model has shown that MSCs were more likely to home to the lung metastatic site than to the primary tumour [78]. A study suggests that tumour cells do not always leave the primary site as single cells but also as ‘heterotypic tumour fragments’ consisting of the metastatic cancer cells along with tumour stromal cells [32]. These clusters of cells were found to migrate to the metastatic site and promote tumour growth. Moreover, CAFs were found to migrate from the primary tumour to the lung metastatic site in mice [32]. Additionally, a study by Kaplan and colleagues using mouse models found that VEGF receptor (VEGFR1) expressing bone marrow derived cells migrated to and formed clusters in pre-metastatic sites before the arrival of tumour cells. Interestingly, blocking VEGFR1 function prevented cluster formation and metastasis [79]. These studies indicate a potential role for bone marrow derived cells in the creation and possibly the maintenance of a metastatic niche.

### Role in the promotion of EMT

The presence of MSCs in the tumour stroma may stimulate EMT of the cancer cells. Research has shown that direct co-culture of breast or gastric cancer cells with human bone marrow derived MSCs resulted in the upregulation of EMT markers N-cadherin, vimentin, Twist and Snail and the downregulation of E-cadherin [80, 81]. Correspondingly, it was found that human MSCs pretreated with TNF-α and IFN-γ, secreted increased levels of TGF-β. Hepatocellular carcinoma cells grown in conditioned medium from the TNF-α and IFN-γ treated MSCs showed marked changes in molecular markers and functional characteristics associated with EMT, such as increased migration and invasion both in vitro and in vivo [82].

### Role in the establishment of distant metastasis

A study by Karnoub and colleagues investigated the effect of MSCs on breast cancer cell motility and migration to the site of metastasis [26]. Human bone marrow derived MSCs were co-injected with the breast cancer cell line, MDA-MB-231, into mice. The chemokine CCL5 was secreted by MSCs, which in turn interacted with its receptor CCR5 on the breast cancer cells, resulting in increased metastasis to the lung [26]. Further strengthening these results, studies were published demonstrating the secretion of CCL5 by in vitro by human bone marrow derived MSCs in response to osteosarcoma cells [83] and breast cancer cells [84]. Additionally, it was found that the release of osteopontin (OPN) by tumour cells induced the production of CCL5 by MSCs, which in turn promoted CCR5 mediated breast cancer cell metastasis (see Fig. 1). Furthermore, MSCs isolated from the site of metastasis (the lung and liver) expressed the CAF markers α-SMA, SDF-1α, TN-C, MMP-2 and MMP-9 [84].

OPN is a chemoattractant with adhesive properties and can facilitate invasion through the binding of integrins, mainly α_v_β_1_, α_v_β_3_, α_v_β_5_, α_v_β_6_, α_8_β_1_ and α_5_β_1,_ on many cell types [85–88]. Increased OPN levels were found to be correlated with prostate cancer progression and an indicator of the presence of distant metastases [89–92]. OPN deficient mice when injected with B16 melanoma cells developed decreased bone metastasis in comparison to wild-type mice [93]. OPN facilitates osteoclastogenesis by mediating osteoclast motility and anchorage to the bone mineral matrix [94–98]. Changes in OPN production within the bone marrow could therefore disrupt bone homeostasis as expression of OPN in breast cancer has been found to be associated with osteolytic bone metastasis [99, 100].

### MSCs at the bone metastatic site

MSCs are bone marrow resident cells and given the poor prognosis in patients diagnosed with metastatic bone cancer, it is a key area in which to explore their role [101]. MSCs play a crucial supportive role for HSCs and their interaction with the surrounding microenvironment maintains a balance between bone formation and resorption. Given the plethora of studies showing the tumour promoting effect of MSC-tumour cell interaction, it is likely that tumour cell infiltration into the bone marrow will have a considerable impact on bone marrow homeostasis.

Entry of cancer cells into the bone marrow may be facilitated by MSCs through adherence of the metastatic cell to bone marrow ECs [33]. Several studies have found that the chemoattraction of tumour cells to the bone marrow is stimulated by bone marrow stromal cell production of SDF-1α (see Fig. 1) [33, 102, 103]. Prostate cancer cells were found to express the receptor CXCR4 and migrate and invade in response to SDF-1α [104, 105]. Human bone marrow derived MSCs were found to promote the transmigration of breast cancer cell lines (MCF7 and T47D) across bone marrow ECs [33]. Tac1 expression in the breast cancer cell lines was found to play a key role in bone marrow EC transmigration and the adherence of the metastatic cells to MSCs through the regulation of CXCR4 and SDF-1α production in the breast cancer cells [33].

Cells of the bone marrow including HSCs, megakaryocytes, macrophages and myeloid-derived suppressor cells have been implicated in developing a hospitable metastatic niche [106]. However, given the plasticity of MSCs and their role in bone remodelling it seems likely that the establishment of tumour cells within the bone marrow would result in cellular cross-talk that would disrupt bone homeostasis. Bone morphogenic protein-4 (BMP-4) within the bone marrow has been shown to stimulate the production of sonic hedgehog (SHH) in prostate cancer LNCaP cells which enhanced BMP-responsive reporter signalling in the mouse stromal cell line, MC3T3-E1, leading to increased osteoblastic differentiation [107].

An interesting study by Joseph et al. investigated the interaction between HSCs derived from the bone marrow of mice implanted with prostate cancer cell lines that formed either osteoblastic or osteolytic metastatic lesions. They found that HSCs derived from the mice with osteoblastic lesions stimulated osteoblastic differentiation of MSCs through BMP2 signalling, while HSCs derived from mice with osteolytic lesions enhanced the differentiation of mixed marrow mononuclear to osteoclasts through IL-6 signalling [108]. It is thought provoking research and the field would benefit from a similar study in which MSCs are isolated from both osteoclastic and osteoblastic metastatic lesions. A better understanding of the impact of tumour cell infiltration on the bone marrow resident cells could reveal better therapeutic targets. The other question is whether these effects are lasting, and if depletion of tumour cells from the metastatic site leaves behind a dysfunctional, destructive microenvironment.

IL-6 is a pro-inflammatory cytokine that is known to mediate cell proliferation, cell survival and lymphocyte differentiation [109]. IL-6 may have an important role in cross-talk within the tumour associated bone marrow microenvironment. Production of IL-6 in multiple myeloma by bone marrow stromal cells induces tumour cell adhesion and osteoclastogenesis [110, 111]. IL-6 secretion in MSCs was found to be stimulated by neuroblastoma cells within the bone marrow which in turn activated osteoclasts [112]. IL-6 was also found to act on neuroblastoma and multiple myeloma cells within the bone marrow by increasing cell proliferation and survival through activation of the signal transducer and activator of transcription 3 (STAT3) pathway [112, 113].

## Conclusions

It is now understood that MSCs interact with and influence tumour cells at various stages of progression. It is not clear however, whether the effect is predominantly tumour promoting or suppressive. Explanations that could account for the conflicting results include differences in experimental design, the heterogeneity within the MSC population or varying responses dependent on the stimuli (explored more extensively in a review by Klopp et al. [114]). Nonetheless, there is extensive evidence to suggest that MSCs can promote tumour growth and drive metastatic progression. Despite this, MSCs are increasingly being studied for their potential in a range of different clinical therapies. It is therefore imperative to understand how they communicate with tumour cells and within the tumour stroma. Given the plasticity of MSCs, future research should consider whether they are reprogrammed at the site of the tumour or if they exert their effects solely through paracrine signalling and direct cell-cell contact. It would also be interesting to ascertain whether there are phenotypic differences in MSCs that are isolated from different tumour types and whether the MSC responds to the tumour according to its stage of progression.

Cancer therapies classically target tumour cells yet, what remains is an activated stroma that provides an encouraging microenvironment for any surviving tumour cells. Evidence to support this comes from studies in breast cancer in which stromal-related gene expression or gene signatures was predictive of clinical outcome [115, 116]. Moreover, pre-treatment of MSCs to concentrations of cisplatin which were toxic to breast cancer cells but not MSCs in vitro was found induce changes in kinase phosphorylation and increased cytokine production in the MSCs and co-culture with breast cancer cells lead to chemoresistance in the tumour cells [117]. It would therefore be of therapeutic interest to investigate the contribution of tumour stromal cells to cancer progression and their activity following cytotoxic treatment.

## Abbreviations

BMP-4: Bone morphogenic protein-4
CAF: Cancer associated fibroblast
CCL5: Chemokine (C-C motif) ligand 5
CCR5: C-C chemokine receptor type 5
EC: Endothelial cell
EMT: Epithelial-to-mesenchymal transition
FSP1: Fibroblast specific protein 1
HSC: Haematopoietic stem cell
IFN-γ: Interferon gamma
IL: Interleukin
MSC: Mesenchymal stem cell
OPN: Osteopontin
SDF-1α: Stromal cell-derived factor 1
SHH: Sonic hedgehog
STAT3: Signal transducer and activator of transcription 3
TAM: Tumour associated macrophage
TGFβ: Transforming growth factor β
TLR: Toll-like receptor
TN-C: Tenascin C
TNF-α: Tumour necrosis factor-α
VEGF: Vascular endothelial growth factor
αSMA: α smooth muscle actin
